# Holy Smoke in Medieval Funerary Rites: Chemical Fingerprints of Frankincense in Southern Belgian Incense Burners

**DOI:** 10.1371/journal.pone.0113142

**Published:** 2014-11-12

**Authors:** Jan Baeten, Koen Deforce, Sophie Challe, Dirk De Vos, Patrick Degryse

**Affiliations:** 1 Center for Surface Chemistry and Catalysis, KU Leuven, Leuven, Belgium; 2 Centre for Archaeological Sciences, KU Leuven, Leuven, Belgium; 3 OD Earth and History of Life, Royal Belgian Institute of Natural Sciences, Brussels, Belgium; 4 Service public de Wallonie, Direction de l’Archéologie, Jambes, Belgium; Université de Poitiers, France

## Abstract

Frankincense, the oleogum resin from *Boswellia* sp., has been an early luxury good in both Western and Eastern societies and is particularly used in Christian funerary and liturgical rites. The scant grave goods in late medieval burials comprise laterally perforated pottery vessels which are usually filled with charcoal. They occur in most regions of western Europe and are interpreted as incense burners but have never been investigated with advanced analytical techniques. We herein present chemical and anthracological results on perforated funerary pots from 4 Wallonian sites dating to the 12–14^th^ century AD. Chromatographic and mass spectrometric analysis of lipid extracts of the ancient residues and comparison with extracts from four *Boswellia* species clearly evidence the presence of degraded frankincense in the former, based on characteristic triterpenoids, *viz.* boswellic and tirucallic acids, and their myriad dehydrated and oxygenated derivatives. Cembrane-type diterpenoids indicate *B. sacra* (southern Arabia) and *B. serrata* (India) as possible botanical origins. Furthermore, traces of juniper and possibly pine tar demonstrate that small amounts of locally available fragrances were mixed with frankincense, most likely to reduce its cost. Additionally, markers of ruminant fats in one sample from a domestic context indicate that this vessel was used for food preparation. Anthracological analysis demonstrates that the charcoal was used as fuel only and that no fragrant wood species were burned. The chars derived from local woody plants and were most likely recovered from domestic fires. Furthermore, vessel recycling is indicated by both contextual and biomarker evidence. The results shed a new light on funerary practices in the Middle Ages and at the same time reveal useful insights into the chemistry of burned frankincense. The discovery of novel biomarkers, namely Δ^2^-boswellic acids and a series of polyunsaturated and aromatic hydrocarbons, demonstrates the high potential for organic chemical analyses of incense residues.

## Introduction

Frankincense, or *olibanum*, is an oleogum resin that exudes in pale yellow to red tears from incisions in the bark of certain *Boswellia* trees (Burseraceae family) thriving in arid regions in the horn of Africa and southern Arabia [1]. It is generally composed of 5–9% essential oil, 65–85% alcohol-soluble resin and the remaining water-soluble gums [2]. The precise chemical composition depends on the botanical species. Most important species are *Boswellia serrata* (India), *B. sacra* (Yemen, Oman), *B. carterii* (Somalia, contentiously considered the same species as *B. sacra*, *e.g.* [3]), *B. papyrifera* (Eritrea, Sudan, Ethiopia) and *B. frereana* (Somalia) [2], [4]. Myrrh is another classical incense source and has often been confused with frankincense. Both oleogum resins have often been loosely designated by the term “incense”, particularly in older literature, generating ambiguity as to the exact taxon [2], [4]. However, whilst frankincense and myrrh trees both belong to the same Burseraceae family and grow in the same regions (*cf. supra*), they constitute two separate genera, *viz. Boswellia* and *Commiphora*, respectively, and their resins have disparate chemical compositions [1]. Plumes of burning frankincense are associated with perfumes, embalming and religious rituals. Furthermore, its medicinal properties attract much attention nowadays as they did in antiquity [5]–[7] but the emission of toxic polyaromatic hydrocarbons (PAHs) during incense burning raises some health concerns as well [8], [9].

The use of incense has a long history. From the late 4^th^ millennium BC onwards, Arabian incense burners began to appear and Egyptians travelled great distances to import frankincense and myrrh [5], [10], [11]. Frankincense was also highly esteemed throughout Assyria, Babylonia, Persia, Greece and the demand reached its peak when Romans burned it in temples, at funerals or in domestic contexts for propitiating the gods [11]. With the spread of Christianity, the incense trade partially collapsed. Early Christians initially repudiated incense burning for its idolatrous connotation but later adopted the use of incense in their rituals [12]–[15]. Trade connections and frankincense consumption, however, never reached the level of Roman times again and this coincided with severe droughts, over-grazing and an increasing need for firewood causing the habitat of *Boswellia* trees in South Arabia to shrink [4], [16]. Unfortunately, our knowledge on how the trade evolved throughout the Middle Ages is rather scattered. Political and religious changes in the Arabian Peninsula brought about shifts in the directions of trading links. Classical incense ports such as Qana’ disappeared and new ones such as al-Shi
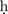
r, Sharma and al-Mukallā began to flourish [16]–[18]. In the 13^th^ and 14^th^ century, Marco Polo mentioned that frankincense trees grew in Shi
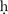
r and in 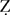
ufār, with those of Shi
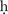
r producing the best quality, and Ibn Battuta recorded great quantities in Hāsik [4]. Recent studies on South Arabian incense burners and resinous remains called for a renewal of interest in medieval incense trade networks [17], [19].

Archaeological frankincense, despite its high value and widespread use, has rarely been identified by chemical analyses. To date, the resin has been demonstrated in remains from sites in Egypt [20]–[22], Yemen [19], [23], [24] and France [25]. These cases, however, represent analyses of resin-like residues. To the best of our knowledge, remains from incense burning have not extensively been characterized. Noteworthy is Basar’s experimental work [26] on pyrolysed frankincense which was aimed at assessing the fate of di- and triterpenoid constituents. Still, archaeological residues, particularly those associated with ceramics, are expected to be more complex due to degradation processes during heating or burial which may be catalyzed by metal ions in the ceramic fabric [27].

The current paper focuses on late medieval funerary pots from the southern Belgian region of Wallonia. They were found in either male or female burials in association with ecclesiastical buildings such as parish, abbey and convent churches, cathedrals, college churches and chapels. Burial types include brick or stone lined graves, graves without lining and wooden coffins. The pots typically date between the 11^th^ and 15^th^ century AD and also occur in northern Spain, France, northern Italy, southern Belgium, Denmark and a few rare sites in England, Scotland and northern Germany. No finds are known from northern Belgium, the Netherlands, northeastern France or southern Germany [28]–[33]. A study of 192 funerary pots from southern Belgian contexts indicates that both the number and position of these funerary pots show much variation between graves. They do not occur systematically, however, and finds remain relatively rare, *i.e.* maximally 1% of all excavated graves at large burial sites contain funerary pots [31], [32], [34]. The vessels are mostly ordinary domestic ceramics like cooking pots or jugs [31]–[33]. Sometimes minor traces of use and chipping are observed which, in the latter case, may indicate that the ceramics were of second choice [31], [32]. Almost all of the pots show lateral perforations which, in most cases, have been made after the pots had been fired [31], [35]. The majority of the pots are filled with charcoal. Vessels from northwestern Europe, including the ones from Belgium, are generally interpreted as censers, based on their appearance and on historic manuscripts and iconographic sources [30], [32]. Vessels from other regions in Europe that lack perforations and charcoal are interpreted as holy water containers or lamps but these were not found in southern Belgium [28], [29], [33], [35].

During a recent study of several funerary pots from multiple sites in southern Belgium, visible residues were observed on the inner side of some of the pots. The results presented herein describe gas chromatography-mass spectrometry (GC-MS) and anthracological analyses aimed at characterizing the type of resin used for incense burning, assessing the potential use of fragrant woody taxa and identifying the type of charcoal fuel. Commercial resins from four *Boswellia* species were analysed for comparison and to verify species-specific criteria. Furthermore, residues absorbed inside the ceramic fabric were also analyzed to find out whether the pots were primarily made for this use or were recycled.

## Materials and Methods

### 1. Solvents, reagents and commercial Boswellia resins

All solvents and reagents used were of analytical or chromatographic grade. Chloroform, *N*,*O*-bis(trimethylsilyl)trifluoroacetamide +1% trimethylchlorosilane (BSTFA+1%TMCS), hexane and *n*-heptadecane, used as internal standard, were purchased from Acros Organics. Methanol and toluene were purchased from Fisher Scientific. The Burseraceae resins *Boswellia papyrifera* Hochst. (“Eritrea 1^st^ choice”), *Boswellia carterii* Birdw. (“Somalia 1^st^ choice”), *Boswellia sacra* Flück. (“Oman white No. 1”), *Boswellia serrata* Roxb. (“Indian siftings”) were obtained from Gerhard Eggebrecht (Süderau, Germany).

### 2. Ethics statement

No permits were required for the described study, which complied with all relevant regulations. The ceramic vessels of this study all come from sites under supervision of archaeologists of the Service Public de Wallonie (SPW) (http://dgo4.spw.wallonie.be/dgatlp). The latter is the authorizing institution of the Walloon region of Belgium and is thus the rightful owner of the vessels. Sophie Challe, co-author of this paper, is ceramologist of the SPW and is therefore legally appointed to coordinate this study. The archaeobotanical and archaeozoological analysis, conducted by Sidonie Preiss and Quentin Goffette at the Royal Belgian Institute of Natural Sciences, were also paid by the SPW. Therefore, no specific permits were required for the publication of this research. In case any results of this research are used for objectives other than publication, all rights are reserved by the DGO4 department (Patrimoine - Direction de l’archéologie) of the SPW. Specimen numbers are listed in Table 1. All vessels belong to the repository of the SPW.

**Table 1:** List of perforated pots with their number of perforations, provenance, age and type of analysis performed.

Ref. Nr.	Site^a^	InventoryNumber	Nr. ofperforations	Vessel type	Burial type	Location	Age	Charcoalanalysis	Residueanalysis^b^
R1	Rebecq	F157	7	Ov	GV	front tower	12^th^–early 13^th^ century	x	–
R2	Rebecq	F174	n.d.	Ov	GV	front tower	12^th^–early 13^th^ century	x	S + A
R3	Rebecq	F178	5	Pc	P	nave	12^th^–early 13^th^ century	x	–
R4	Rebecq	F186	4	Pc	P	nave	12^th^–early 13^th^ century	x	–
R5	Rebecq	F187	4	Pc	P	nave	12^th^–early 13^th^ century	x	–
R6	Rebecq	F188	4	Pc	P	nave	12^th^–early 13^th^ century	x	–
R7	Rebecq	F189	4	Pc	P	nave	12^th^–early 13^th^ century	x	–
R8	Rebecq	F190	>3	Pc	P	nave	12^th^–early 13^th^ century	x	–
R9	Rebecq	F220	n.d.	Pc	P	nave	12^th^–early 13^th^ century	x	S + A
Q10	Quaregnon	F317 US 02.485	6	Pc	GV	central nave	13^th^–14^th^ century	–	S
Q11	Quaregnon	F284 US 02.359-n°171	5	Ov	GV	central nave	13^th^–14^th^ century	–	F
Q12	Quaregnon	F284 US 02.359-n°172	5	Ov	GV	central nave	13^th^–14^th^ century	–	F
N13	Namur	01.347.0054	>2	Ov	CP	fill	13^th^–14^th^ century	–	S + A
L14	Liège	T156 Fi 537	3	Pc	UG	chapel of Saint-Luc	late 13^th^–early 14^th^ century	x	S
L15	Liège	T156 Fi 2760	4	Pc	UG	chapel of Saint-Luc	late 13^th^–early 14^th^ century	–	S
L16	Liège	T183 Fi 1011/1	4	Pc	GV	southeastern wing	late 13^th^–early 14^th^ century	x	–
L17	Liège	T183 Fi 1011/2	4	Pc	GV	southeastern wing	late 13^th^–early 14^th^ century	x	–
H18	Huy	F01.151 01.169.2	0	Ov	UG	1st central bay	late 13^th^–14^th^ century	x	–
H19	Huy	F01.151 01.169.3	0	Ov	UG	1st central bay	late 13^th^–14^th^ century	x	–
H20	Huy	F01.48 01.061.1	0	Ov	UG	5th bay nord	late 13^th^–14^th^ century	x	–
H21	Huy	F01.48 01.154.1	0	Ov	UG	2nd central bay	late 13^th^–14^th^ century	x	–
H22	Huy	F01.48 1 159	0	Ov	UG	1st bay nord	late 13^th^–14^th^ century	x	–
H23	Huy	F01.48 01.161.1	0	Ov	UG	1st bay nord	late 13^th^–14^th^ century	x	–
H24	Huy	F01.48 01.161.3	n.d.	Ov	UG	1st bay nord	late 13^th^–14^th^ century	x	–
H25	Huy	F01.48 1 195	>1	Ov	UG	5th bay southern nave	late 13^th^–14^th^ century	x	–
H26	Huy	F01.48 1 164	2	Ov	UG	not determined	late 13^th^–14^th^ century	x	–

Abbreviations: n.d. = not determined, Ov = ovoid pot, Pc = pitcher, CP = cesspit, GV = grave, P = pit, UG = unlined grave.

a Burial locations: Rebecq = parish church of Saint-Géry, Quaregnon = parish church of Saint-Quentin, Namur = Place Maurice Servais, Liège = western cloister of the Notre-Dame-et-Saint-Lambert cathedral, Huy = parish church of Saint-Hilaire.

b Types of residues sampled: S = surface residue, A = absorbed residues in ceramic, F = vessel filling.

### 3. Archaeological materials

**Figure 1:**
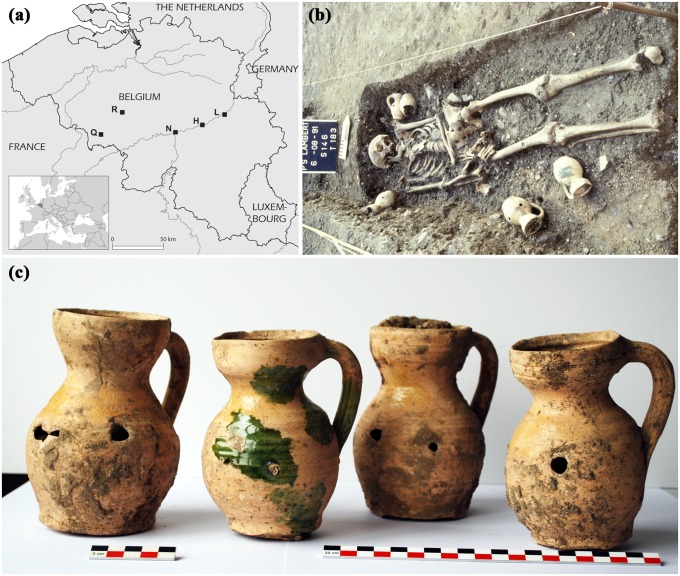
Perforated funerary pots and their context: (a) location of archaeological sites in Belgium, (b) picture of grave T183 with funerary pots (Liège, Place Saint-Lambert), (c) picture of perforated funerary pots from grave T183 (Liège, Place Saint-Lambert). Abbreviations: H = Huy, L = Liège, N = Namur, Q = Quaregnon, R = Rebecq.

Of the 192 perforated pots that were uncovered from Belgian burials, 26 pots from 4 different sites have been selected for this study (Figure 1, Table 1). All vessels are dated to the 12^th^–14^th^ century. The samples from Rebecq originate from two ovoid pots (R1–R2) from a tomb in the front tower of the parish church Saint-Géry of Rebecq and from 7 pitchers (R3–R9) recovered from a pit in the nave of the same church [36]. This pit, from which a total of 8 pitchers were recovered, does not seem to be related to any of the surrounding burials, but might be related to the inauguration of the church. One pitcher (Q10) and two globular pots (Q11–Q12) from two different graves excavated in the central nave of the parish church of Saint-Quentin in Quaregnon have been studied [37]. The ovoid pot (N13) from Namur is the only one that does not originate from a religious site as it has been recovered from the fill of a cesspit from an urban domestic site at the Maurice Servais square [38]. Four pitchers (L14–L17) from excavations at the Place Saint-Lambert in Liège were recovered from the cloister attached to the Notre-Dame-et-Saint-Lambert cathedral; two from an unlined grave in the chapel of Saint-Luc and another two from a grave that was excavated in the southeastern wing of the cloister [39]. The nine ovoid pots (H18–H26) from Huy that have been studied originate from unlined graves from the Saint-Hilaire church [40]. Except for 6 pots from Huy (H18–H23) which did not show perforations, all pots were perforated after they had been fired. Some of the pots showed evidence for surface treatment on the outer walls (cf. Figure 1), but not on the inner walls.

### 4. Lipid extraction

Residues from 8 unwashed pots have been studied (Table 1). Surface residues (100–1000 mg) were sampled with a spatula or a hand drill. All residue types were crushed with mortar and pestle. The sherd samples (ca. 5 g) were further powdered in a ball mill (stainless steel). A standard lipid extraction was performed [27] using chloroform: methanol (2∶1 v/v) as solvent and ultrasonication to assist the extraction. 50 µg of *n*-heptadecane was added as internal standard prior to extraction. After centrifugation and filtration (PTFE 0.45 µm, Macherey-Nagel), the extract was concentrated under a gentle stream of nitrogen and derivatised with BSTFA+1%TMCS (60°C, 60 min) and dissolved in toluene before analysis with GC-MS. The commercial *Boswellia* resins were extracted and derivatised in the same manner.

### 5. Gas Chromatography Mass Spectrometry

GC-MS analyses were carried out using a 7890A Agilent gas chromatograph coupled to a 5977A mass spectrometric detector. The GC was equipped with a HP-5MS capillary column (30 m×0.25 mm×0.25 µm). 1 µl of each sample was injected using splitless (head pressure 9.15 psi) or pulsed splitless (head pressure 20 psi) injection at a temperature of 250°C. The initial oven temperature of 80°C was held for 1 min, ramped at 10°C min^−1^ to 150°C, then ramped at 4°C min^−1^ to 320°C and finally kept at this temperature for 20 min. The transfer line and ion source were held at 330°C and 230°C, respectively. Mass spectra were taken between masses *m*/*z* 50–700 with an ionization potential of 70 eV. Peak identifications were performed using the NIST11 mass spectral database, published mass spectra, retention characteristics (*viz.* comparison to reference *Boswellia* extracts, published retention indices), mass spectral deconvolution (using Masshunter and AMDIS software) and interpretation of mass spectra [20], [26].

### 6. Anthracological analyses

Charcoal from the content of 21 pots has been studied (Table 1). From each of these pots, a minimum of 100 charcoal fragments has been identified. If fewer charcoal fragments were present, all of these have been studied. For identification, each fragment was manually broken along three different planes (transversal, radial, tangential). The anatomical characteristics visible on these fresh surfaces were studied using reflected light microscopy (50–500x) and wood anatomical atlases [41]–[44] and a reference collection of modern charred wood species.

## Results

### 1. GC-MS analysis of modern Boswellia resins

**Figure 2:**
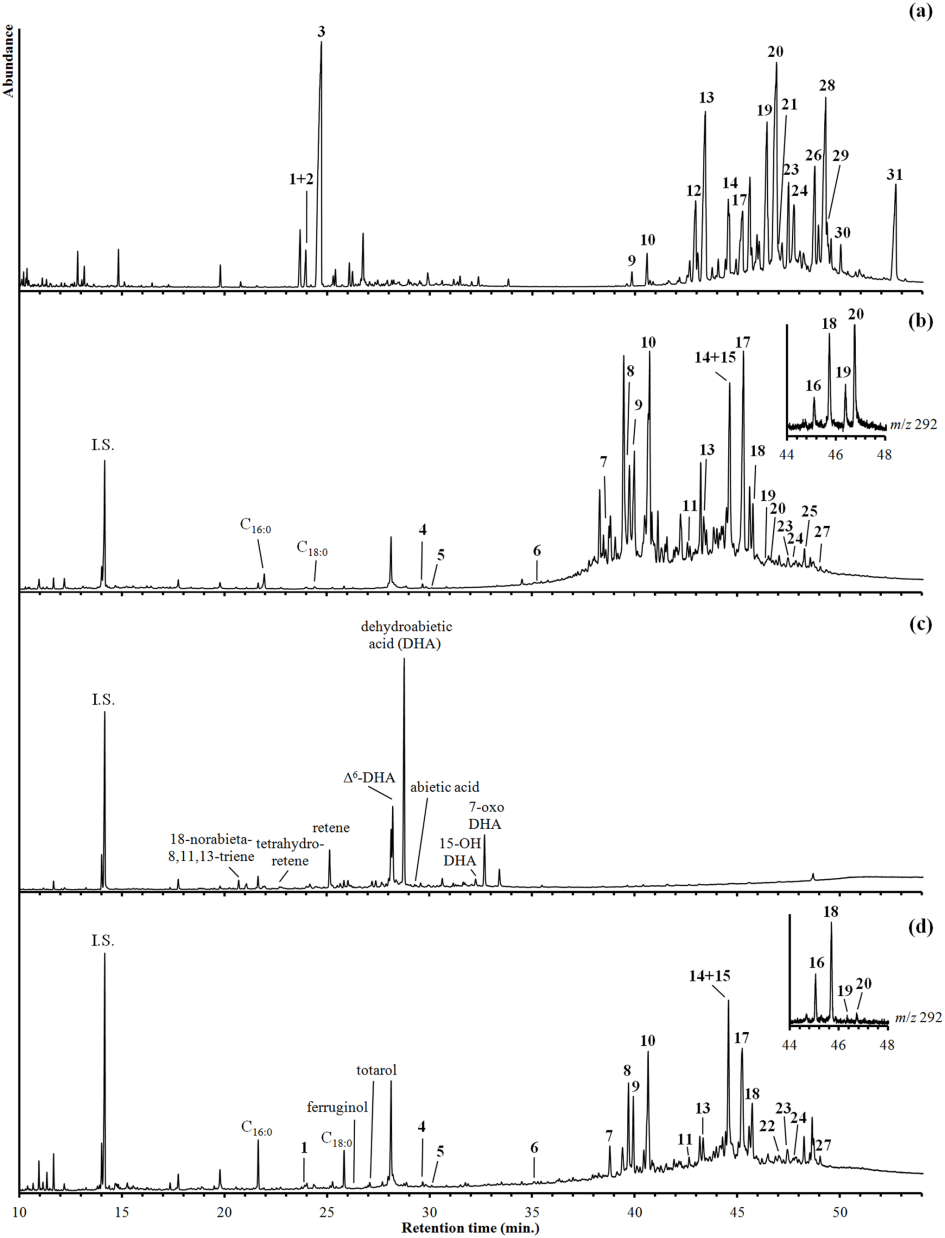
GC-MS chromatograms of silylated extracts of (a) modern *Boswellia carterii* resin, (b) sample R9, (c) sample Q10 and (d) sample Q11. Insets in (b) and (d) show partial *m*/*z* 292 chromatograms. Legend to compound labels: I.S. = internal standard, C16∶0 = palmitic acid, C18∶0 = stearic acid, DHA = dehydroabietic acid, 1 = serratol (free OH), 2 = incensol (free OH), 3 = incensol (OTMS), 4 = des-A-ursa-5(10),12-diene, 5 = des-A-26,27-dinorursa-5,7,9,11,13-pentaene, 6 = 1,9-dimethylchrysene, 7 = 24-noroleana-3,9(11),12-triene, 8 = 24-norursa-3,9(11),12-triene, 9 = 24-noroleana-3,12-diene, 10 = 24-norursa-3,12-diene, 11 = 24,25,26,27-tetranorursa-1,3,5(10),6,8,11,13-heptaene, 12 = *epi*-β-amyrin, 13 = *epi*-α-amyrin, 14 = β-amyrenone, 15 = 24-norursa-3,12-dien-11-one, 16 = Δ^2^-α-boswellic acid, 17 = α-amyrenone, 18 = Δ^2^-β-boswellic acid, 19 = α-boswellic acid, 20 = β-boswellic acid, 21 = lupeolic acid, 22 = 2,9-dimethylpicene, 23 = β-elemonic acid, 24 = β-elemolic acid, 25 = 11-keto-α-amyrin, 26 = 3-*O*-acetyl-α-boswellic acid, 27 = 11-keto- Δ^2^-β-boswellic acid, 28 = 3-*O*-acetyl-β-boswellic acid, 29 = 3-*O*-acetyl-lupeolic acid, 30 = 11-keto-β-boswellic acid, 31 = 3-*O*-acetyl-11-keto-β-boswellic acid.

Commercial resins were analysed to establish a database of mass spectra, to compare the composition with the archaeological samples, and to verify species-specific criteria. Only di- and triterpenoids are reported here and the results are summarized in Table 2 (for molecular structures, see Figure S1). Volatile mono- and sesquiterpenoids are also detected but were not investigated in detail because they are not expected to be preserved in residues of incense burning. Major triterpenoids in all species are boswellic acids and their 3-*O*-acetyl derivatives with a clear ursane over oleanane predominance (Figure 2, Table 2). Oxygenated forms of boswellic acids are also detected, *e.g.* 11-keto-β-boswellic acid, 11-hydroxy-β-boswellic acid and their corresponding 3-*O*-acetyl derivatives. Boswellic acids and their derivatives are specific for *Boswellia* species, particularly *B. carterii*, *B. sacra*, *B papyrifera* and *B. serrata* [1], [22], [26]. The ratio of 3-*O*-acetyl-11-keto-β-boswellic acid to 11-keto-β-boswellic acid has been proposed as a further species-specific criterium [45], [46]. It amounts to 1 for *B. serrata* and 4–7 in *B. sacra*, *B. carterii* and *B. papyrifera*. Tirucallol and tirucallic acids such as β-elemonic acid, β-elemolic acid and β-elemolic acid acetate are also present and their relative abundance is higher in *B. serrata* and *B. papyrifera* than in *B. sacra* and *B. carterii*. A reversed pattern is observed for amyrins and lupanes which are most abundant in *B. sacra* and *B. carterii* (Table 2).

**Table 2:** Relative abundances (%) of di- and triterpenoids identified in commercial specimens of *Boswellia* resins.

Compound classes	CommonName	SystematicName	Type	*Boswellia* *carterii*	*Boswellia* *sacra*	*Boswellia* *serrata*	*Boswellia* *papyrifera*
**Diterpenoids**	cembrene A		C	1.0	2.1	1.7	1.8
	cembrene isomer (?)		C	0.2	1.4	0.8	-
	serratol isomer (?)		C	0.2	1.8	0.9	-
	cembrene C		C	0.3	1.7	0.5	0.2
	verticilla-4(20),7,11-triene			-	-	-	6.6
	unknown (?)			2.3	1.0	0.7	0.2
	incensol		C	*17.7*	tr	tr	6.9
	serratol		C	*7.0*	13.7	14.3	-
	incensol acetate		C	-	-	-	11.8
	incensol oxide acetate		C	-	-	-	0.1
**Triterpenoids**	*epi*-β-amyrin	olean-12-en-3α-ol	O	3.6	2.9	0.9	0.1
	*epi*-α-amyrin	urs-12-en-3α-ol	U	10.2	5.0	3.0	0.3
	*epi*-lupeol	lup-20(29)-en-3α-ol	L	3.8	4.5	0.7	0.3
	tirucallol	tirucalla-8,24-dien-3-ol	T	0.2	0.4	0.6	0.4
	β-amyrenone	olean-12-en-3-one	O	*0.5*	*3.8*	*0.5*	ce
	β-amyrin	olean-12-en-3β-ol	O	ce	ce	ce	ce
	α-amyrenone	urs-12-en-3-one	U	*1.0*	*10.8*	*1.1*	*0.5*
	α-amyrin	urs-12-en-3β-ol	U	*2.6*	*3.0*	*1.8*	*0.6*
	lupeol	lup-20(29)-en-3β-ol	L	*0.4*	*1.0*	ce	ce
	α-boswellic acid	3α-hydroxy-olean-12-en-24-oic acid	O	7.6	4.6	9.3	4.8
	β-boswellic acid	3α-hydroxy-urs-12-en-24-oic acid	U	13.5	14.8	23.2	11.3
	lupeolic acid	3α-hydroxy-lup-20(29)-en-24-oicacid	L	*1.1*	*1.5*	*0.3*	*0.1*
	11-hydroxy-β-boswellic acid	3α,11α-dihydroxy-urs-12-en-24-oicacid	U	0.7	-	0.4	0.9
	β-elemonic acid	3-oxo-tirucalla-8,24-dien-21-oicacid	T	2.9	tr	6.0	13.9
	β-elemolic acid	3-hydroxy-tirucalla-8,24-dien-21-oicacid	T	*1.5*	tr	*0.3*	*2.2*
	3-*O*-acetyl-β-elemolic acid	3-acetoxy-tirucalla-8,24-dien-21-oicacid	T	*0.4*	tr	*5.9*	*5.5*
	3-*O*-acetyl-α-boswellic acid	3α-acetoxy-olean-12-en-24-oic acid	O	3.9	6.3	5.5	7.1
	3-*O*-acetyl-β-boswellic acid	3α-acetoxy-urs-12-en-24-oic acid	U	9.3	14.5	18.3	11.2
	3-*O*-acetyl-lupeolic acid	3α-acetoxy-lup-20(29)-en-24-oicacid	L	*1.0*	*2.1*	*0.1*	*0.5*
	3-*O*-acetyl-11-hydroxy-β-boswellic acid	3α-acetoxy-11α-hydroxy-urs-12-en-24-oic acid	U	0.6	tr	0.2	2.2
	11-keto-β-boswellic acid	3α-hydroxy-11-oxo-urs-12-en-24-oicacid	U	0.8	0.4	1.5	2.0
	3-*O*-acetyl-11-keto-β-boswellic acid	3α-acetoxy-11-oxo-urs-12-en-24-oicacid	U	5.6	2.7	1.6	8.3
**Total amyrins: ** ***epi*** **-amyrins (3α-OH),** **amyrenones (3-oxo) and amyrins (3β-OH)**				17.9	25.4	7.3	1.5
**Total boswellic acids: boswellic acids,** **hydroxy/keto boswellic acids and their** **acetates**				42.0	43.3	60.0	47.8
**Total lupanes (L): epi-lupeol, lupeol, lupeolic** **acid and its acetate**				6.3	9.1	1.1	0.9
**Total tirucallanes (T): β-elemonic acid, β-** **elemolic acid and its acetate**				5.0	0.4	12.7	22.1
**Ratio of ursanes (U) to oleananes (O)** a				2.2	2.5	2.8	1.9
**Ratio of 3-** ***O*** **-acetyl-11-keto-β-boswellic acid** **to 11-keto-β-boswellic acid**				6.9	6.4	1.1	4.2

Legend: - = not detected, ce = co-eluting compound which could not be resolved with mass spectral deconvolution, tr = trace, italic numbers are deconvoluted compounds. Structure types: C = cembrane, O = oleanane, U = ursane, L = lupane, T = tirucallane. Major diterpenoid and triterpenoid structures are depicted in figure S1.

a Calculated using *epi*-amyrins, boswellic acids and 3-*O*-acetyl-boswellic acids.

**Figure 3:**
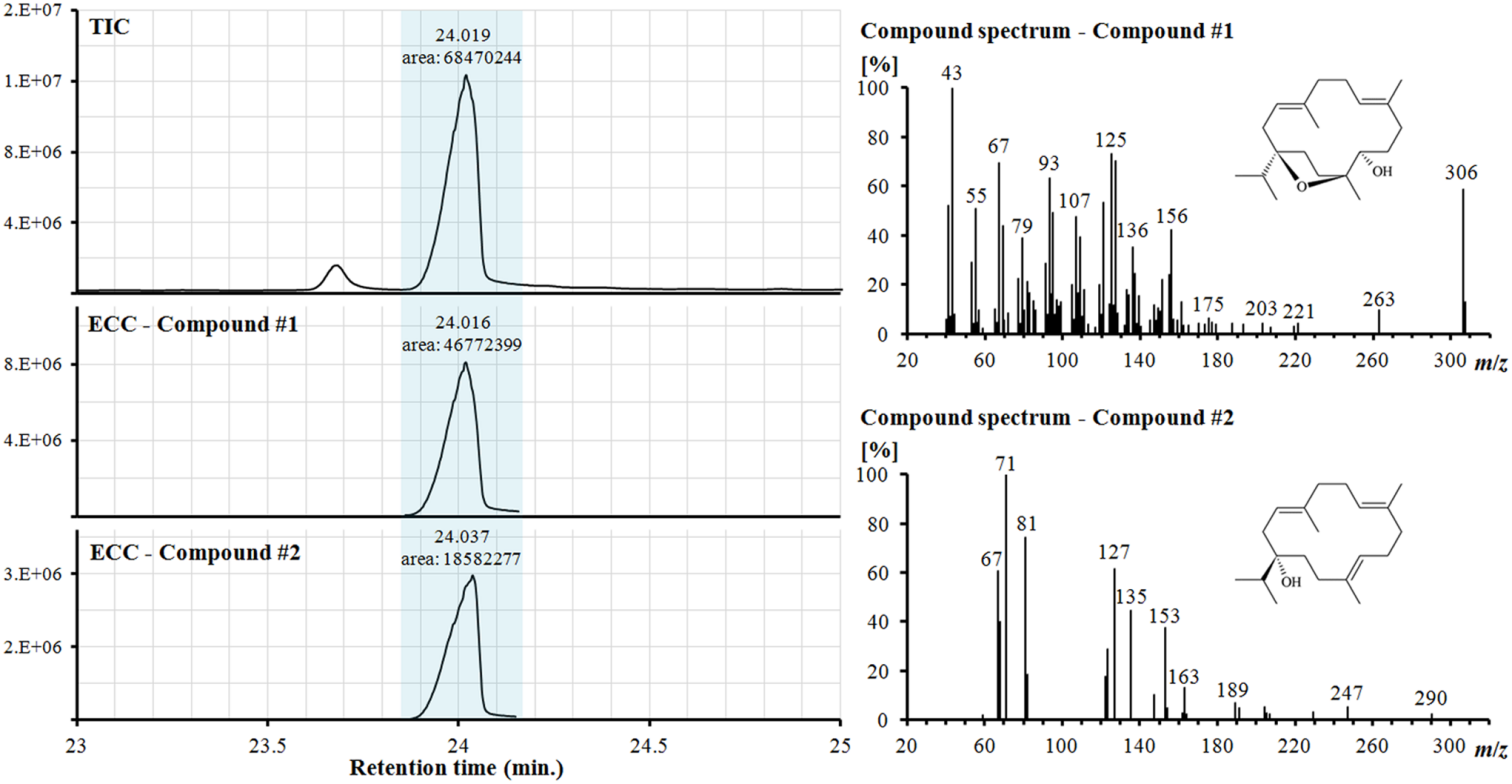
Mass spectral deconvolution of incensol and serratol in an underivatized extract of *Boswellia carterii*. Abbreviations: TIC = total ion count chromatogram, ECC = extracted compound chromatogram, *m*/*z* = mass to charge ratio. Deconvoluted mass spectra are identified as (#1) incensol and (#2) serratol (see text).

The diterpenoid profile consists mainly of cembrane type alcohols such as incensol, serratol and incensol acetate. Incensol and serratol are not fully derivatized (Figure 2) and the free alcohols show almost full coelution (retention indices 2150 and 2152, respectively). This is also evident from the data from Hamm et al. [23], in which compounds 127 and 128 (same retention indices) were identified as incensol and isoincensol co-eluting with isoincensol acetate, respectively, based on mass spectral data. However, comparison of their mass spectra with those of isolated incensol [47] and serratol [48] reveals that compound 127 was correctly identified as incensol but that compound 128 corresponds to serratol, a diterpenoid common to *Boswellia* species, particularly *B. sacra*, *B. carterii* and *B. serrata* [46], [48], [49]. We were able to successfully resolve these co-eluting compounds by mass spectral deconvolution, with the summed peak areas of the extracted compound chromatograms (ECCs) amounting to 95% of the peak area of the total ion count chromatogram (Figure 3). Diterpenoid profiles of the commercial *Boswellia* resins are dominated by incensol and serratol in *B. carterii* (61% and 24% of total identified diterpenoids, respectively), by serratol in *B. sacra* (63%) and *B. serrata* (76%), and by verticilla-4(20),7,11-triene, incensol and incensol acetate in *B. papyrifera* (24%, 25% and 43%, respectively).

### 2. GC-MS analysis of archaeological residues

The surfaces residues and vessel fillings of most perforated pots consist predominantly of ursane- and oleanane-type triterpenoids besides an array of minor aliphatic lipids (e.g. fatty acids, alkanols, alkanes) and synthetic contaminants (e.g. oleamide, phthalates). These minor components are not necessarily related to the use-phases of the pots but may have intruded the residues during prolonged contact with soil particles and during transportation in plastic bags, respectively [50], [51]. To exclude these contamination issues and to verify earlier vessel uses, residues absorbed inside the ceramic fabric were analyzed whenever possible. Contrary to the triterpenoid predominance in most samples, the lipid composition of samples Q10 and N13 was dominated by diterpenoids and aliphatic lipids, respectively. The chromatograms of samples R9, Q10 and Q11 are displayed in Figure 2 and a list of all detected compounds with retention and mass spectral data can be found in Table S1. Major diterpenoid and triterpenoid structures are depicted in Figure S1.

#### 2.1 Terpenoid signatures in samples from Rebecq, Quaregnon and Liège

Major peaks (∼10–100 µg g^−1^) in the chromatograms of surface residues from Rebecq, Quaregnon and Liège are from 24-norursa-3,9(11),12-triene, 24-norursa-3,12-diene, 24-norursa-3,12-dien-11-one, 3-*O*-acetyl-ursa-9(11),12-diene, α-amyrenone, 3-*O*-acetyl-α-amyrin and corresponding oleanane type compounds (Figure 2).

**Figure 4:**
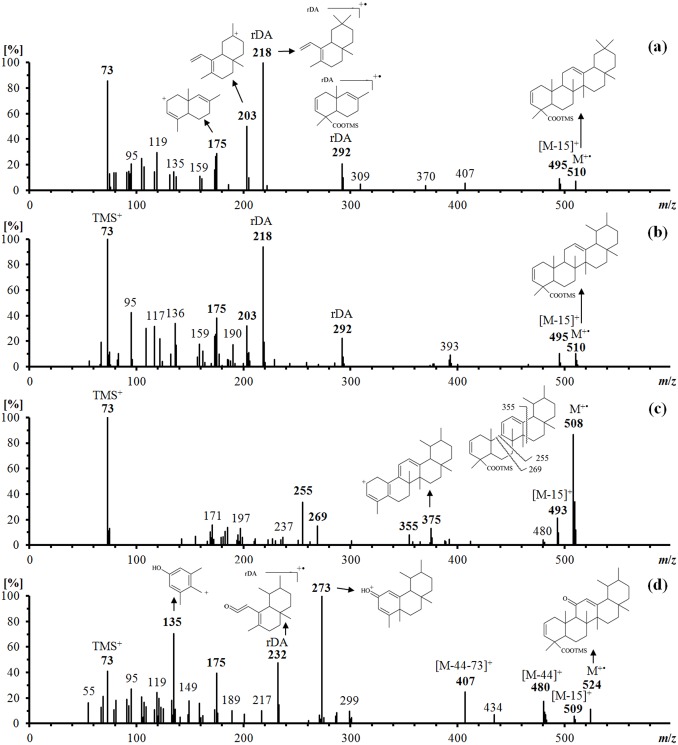
Deconvoluted mass spectra of Δ^2^-boswellic acids as recorded in sample Q11: (a) Δ^2^-α-boswellic acid = oleana-2,12-dien-24-oic acid, (b) Δ^2^-β-boswellic acid = ursa-2,12-dien-24-oic acid, (c) Δ^2,9(11)^-β-boswellic acid = ursa-2,9(11),12-dien-24-oic acid and (d) 11-keto-Δ^2^-β-boswellic acid = 11-keto-ursa-2,12-dien-24-oic acid. Abbreviations: *m*/*z* = mass to charge ratio, TMS = trimethylsilyl, rDA = retro-Diels-Alder, M = molecular ion.

Trace amounts (0.1–0.2 µg g^−1^) of boswellic acids are observed in two samples (R9 and Q11) after selected ion chromatogram screening using the retro-Diels-Alder (rDA) fragment at *m*/*z* 292 and the molecular ion (M^+•^) at *m*/*z* 600 (peak 19 and 20 in insets of Figure 2b and 2d). However, in samples R2, R9, Q11 and Q12, the *m*/*z* 292 ion chromatograms also revealed two clear peaks at slightly earlier retention times, *viz*. peak 16 and 18 in Figure 2. The corresponding peak areas of the total ion chromatograms reveal that they are much more abundant (4.0–7.2 µg g^−1^) than the boswellic acids. Their deconvoluted mass spectra (Figure 4a and 4b) are nearly identical and show clear features of a boswellic acid derivative, *viz*. ions with *m*/*z* 73, 175, 203, 218 and 292 [20], [26]. Peaks at *m*/*z* 495 and *m*/*z* 510 clearly mark the [M–15]^+^ and [M]^+•^ ions, respectively, and enable to identify these compounds as dehydrated (or deacetoxylated) boswellic acids, *i.e.* the alcohol (or acetoxy) group at C-3 has been lost together with a hydrogen at C-2 resulting in the formation a Δ^2^ double bond. The higher intensity of the *m*/*z* 203 ion in the first compound (peak 16, Figure 4a) is diagnostic for an oleanane structure, *viz.* Δ^2^-α-boswellic acid (oleana-2,12-dien-24-oic acid), while the second compound (peak 18, Figure 4b) corresponds to the ursane structure, *viz*. Δ^2^-β-boswellic acid (ursa-2,12-dien-24-oic acid). Additionally, the chromatograms of samples R2, R9, Q11 and Q12 showed smaller peaks of other dehydrated boswellic acid derivatives, namely ursa-2,9(11),12-trien-24-oic acid and 11-keto-ursa-2,12-dien-24-oic acid. The identification of these novel compounds is also based on mass spectral interpretation as presented in Figure 4c and 4d. Further details on mass spectral fragmentation patterns and retention characteristics of all identified Δ^2^-compounds can be found in File S1.

**Figure 5:**
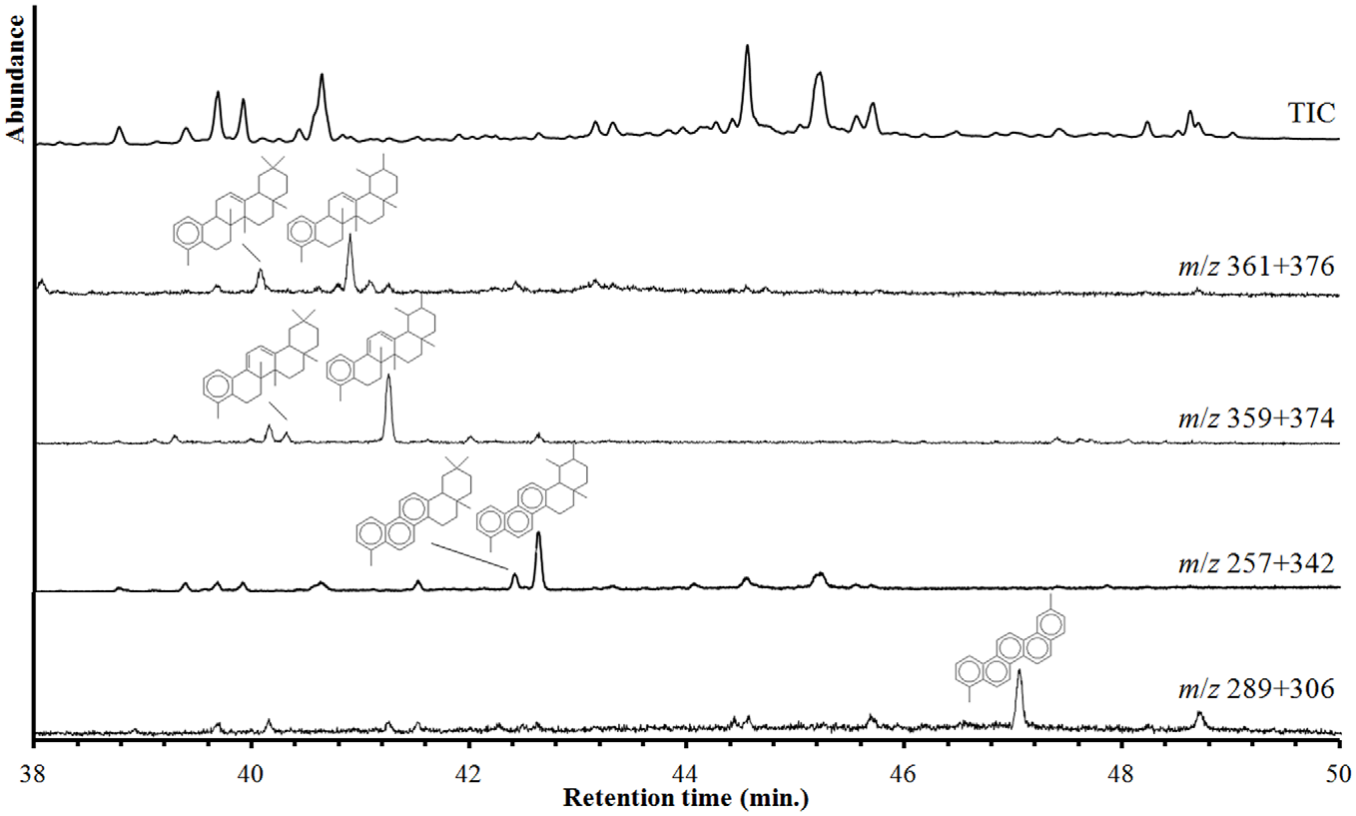
Detail of the total ion count (TIC) and extracted ion chromatograms of sample Q11, showing the presence of mono- and polyaromatic triterpenoids. Selected ions are specific for 24,25-dinorursa-1,3,5(10),12-tetraene (*m*/*z* 361+376), 24,25-dinorursa-1,3,5(10),9(11),12-pentaene (*m*/*z* 359+374), 24,25,26,27-tetranorursa-1,3,5(10),6,8,10,13-heptaene (*m*/*z* 357+342) and corresponding oleanane compounds and 2,9-dimethylpicene (*m*/*z* 289+306).

As stated above, boswellic acids and their derivatives are highly diagnostic for frankincense. Other minor constituents of frankincense are also present, mostly in trace abundances (0.1–0.3 µg g^−1^). These include lupeolic acid (sample R9), its Δ^2^ derivative (sample Q11), tirucallic acids (samples R9 and Q11) and serratol and incensol (samples Q11, L14 and L15). Furthermore, ring A contracted neotriterpenoids (samples R9, Q11, L14 and L15) and polyunsaturated or aromatic hydrocarbons (samples R2, R9, Q11, Q12, L14 and L15) are observed in minor amounts. The latter include tetracyclic hydrocarbons, e.g. des-A-ursa-5(10),12-diene, des-A-26,27-dinorursa-5,7,9,11,13-pentaene and 1,9-dimethylchrysene, as well as pentacyclic hydrocarbons, e.g. 24,25-dinorursa-1,3,5(10),12-tetraene, 24,25,26,27-tetranorursa-1,3,5(10),6,8,10,13-heptaene, 1,2,9-trimethyl-1,2,3,4-tetrahydropicene, 2,9-dimethylpicene. Many of these compounds exhibit significant coelution and their detection and identification was mainly achieved by selected ion chromatogram screening (Figure 5) and by using published mass spectral data and retention characteristics [52]–[57].

The molecular composition of sample Q10 has a deviating pattern and consists exclusively of diterpenoids (Figure 2). Major peaks (30–170 µg g^−1^) were from abietane compounds such as 18-norabieta-8,11,13-triene, tetrahydroretene, retene, 13-isopropyl-5α-podocarpa-6,8,11,13-tetraen-16-oic acid, dehydroabietic acid, 15-hydroxydehydroabietic acid and 7-oxodehydroabietic acid. Pimarane compounds such as isopimara-8,15-dien-8-oic acid, pimaric acid, sandaracopimaric acid, isopimaric acid are also present. These abietane and pimarane diterpenoids are highly diagnostic for a tar derived from the Pinaceae family [58], [59]. In addition, the chromatogram displays trace amounts of 16-nordehydroabietic acid, 16,17-bisnordehydroabietic acid, 7-oxo-18-norabieta-8,11,13-triene, 15,16,17-trisnordehydroabietic acid, simonellite, 5α- and 5β-9,10-secodehydroabietic acid as well as polycyclic aromatic hydrocarbons (PAHs) such as phenanthrene, methylphenanthrenes, pimanthrene, 7-ethyl-1-methylphenanthrene, methylcyclopentenophenanthrene and methylretenes. Dehydroabietic acid, 7-oxodehydroabietic acid, retene and some pimaric acids are also detected in trace amounts in samples R2, R9, Q11, L14 and L15.

Phenolic diterpenes ferruginol, totarol and their corresponding ketones are detected in trace amounts (0.1–0.3 µg g^−1^) in samples Q11 and Q12 (Figure 2). These are highly diagnostic for the Cupressaceae (e.g. *Tetraclinis*, *Juniperus*, *Cupressus*) and Podocarpaceae family (e.g. *Podocarpus*) and also occur in *Cedrus atlantica* [60].

The absorbed residues from samples R2 and R9 contain only trace amounts of triterpenoids such as 24-norursa-3,12-diene, 24-norursa-3,12-dien-11-one, α-amyrenone and corresponding oleanane compounds. No other molecule classes are present.

#### 2.2 Aliphatic signatures in samples from Namur

**Figure 6:**
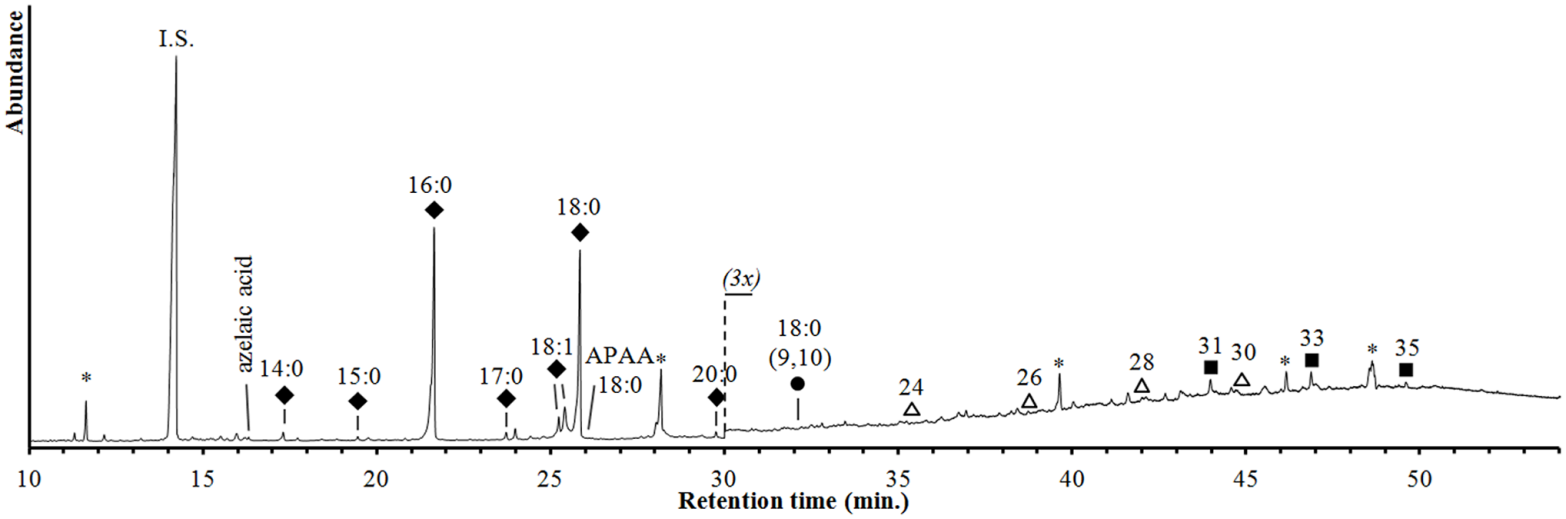
GC-MS chromatogram of silylated extracts of sample N13. Abbreviations (n = carbon number, m = number of double bonds): internal standard (I.S.), ω-(alkylphenyl)-alkanoic acid (APAA, n:m), fatty acids (filled rhombi, n:m), 9,10-dihydroxyalkanoic acid (filled circle, n:m), n-alkanols (open triangles, n) and mid-chain ketones (filled squares, n). Synthetic contaminants are marked with an asterisk.

**Figure 7:**
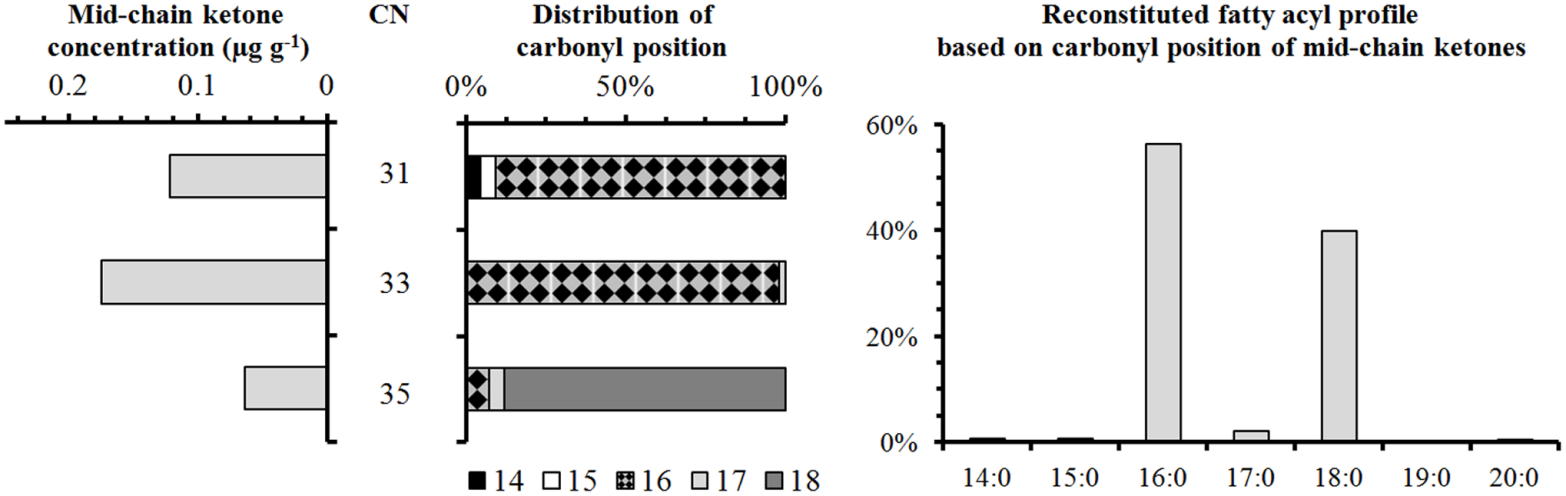
Mass spectral deconvolution of the mid-chain ketones detected in sample N13. Based on the profile of mid-chain ketones and the distribution of their carbonyl position, the fatty acyl profile can be reconstituted (see text). Ketones are abbreviated by their carbon number (CN) and fatty acyl chains are abbreviated by n:m (n = carbon number, m = number of double bonds).

The lipid extracts of both the absorbed and surface residues of sample N13 consist predominantly of aliphatic compounds (Figure 6). Most abundant are palmitic acid (C_16∶0_) and stearic acid (C_18∶0_) exhibiting absolute concentrations of 3.5 µg g^−1^ and 3.0 µg g^−1^, respectively. Although these concentrations are quite low in comparison to food residues from other sites [27] and could possibly be interpreted as background contamination [61], [62], they are detected together with compounds that are typically associated with food processing, e.g. azelaic acid, C_18_ vicinal dihydroxy fatty acids, C_18_ ω-(o-alkylphenyl)alkanoic acids and C_31–35_ mid-chain ketones. These compounds provide unambiguous evidence for the heating of fatty materials in ceramic vessels at temperatures above 300°C [63], [64]. Furthermore, mid-chain ketones, when formed by condensation of saturated fatty acids, can provide information as to the source of the fatty material, based on the distribution of the carbonyl position [27]. Assuming that all ketones arise from acyl lipid pyrolysis, the original acyl distribution can be reconstructed by mass spectral deconvolution (Figure 7). The resulting profile is characterized by a relatively high amount of stearyl moieties and small amounts of C_15_ and C_17_ fatty acyl moieties, which are characteristic features of ruminant fats. Traces of long-chain (C_22_–C_32_) alkanols could be indicative for leafy vegetables although they were only detected in trace abundances (Figure 6).

The lipid profile of the absorbed residues is almost identical to that of the surface residue, except for small peaks of coprostanol, epicoprostanol, cholesterol and sitosterol which are only detected in the surface residue. Especially coprostanol and epicoprostanol are biomarkers for faeces from an omnivore [65]. This is not surprising as the pot was recovered from a latrine.

#### 3. Anthracological analysis

A total of 1854 charcoal fragments has been identified from the content of the different funerary pots, resulting in a minimum number of 11 identified taxa (Table 3). The charcoal assemblage from all the studied funerary pots is dominated by oak (*Quercus* sp.), beech (*Fagus sylvatica*) or hornbeam (*Carpinus betulus*). All identified taxa can have occurred in the vegetation surrounding the sites [66]. No exotic taxa have been found and none of the identified taxa has specific odoriferous or aromatic characteristics.

**Table 3:** Identification and abundance (in %) of charcoal fragments found in the perforated pots.

Botanical species	Rebecq									Liège PSL			Huy								
	R1	R2	R3	R4	R5	R6	R7	R8	R9	L14	L16	L17	H18	H19	H20	H21	H22	H23	H24	H25	H26
*Alnus* sp.										2.0											
*Betula* sp.										9.8			37.7	16.3	11.9	5.6	18.2		2.0	22.3	11.0
*Carpinus betulus*	6.5	54.6									0.9	28.2	25.5		14.9	0.9	7.6	7.1		6.8	8.0
*Corylus avellana*	0.9									15.7			0.9		14.9		6.1				
*Fagus sylvatica*	1.9	0.8	100	100	100	100	100	100	100	50.0	1.9	59.2			3.0	2.8	10.6	28.6	40.6		
*Hedera helix*												1.0									
*Cytisus* type											1.9		17.0			1.9	4.5				1.0
Maloideae	26.9	1.7													6.9						
*Prunus* type *spinosa*															1.0						
*Quercus* sp.	62.0	41.2								14.7	72.6	1.0	11.3	37.2	33.7	64.5	39.4	57.1	55.4	61.2	80.0
*Salix* sp.													4.7	37.2	3.0	5.6	12.1	7.1			
*Salix*/*Populus*											21.7				9.9	16.8				6.8	
bark indet.	1.9	1.7									0.9	4.9	1.9	9.3	1.0	1.9	1.5		1.0	1.9	
indet.										4.9		5.8	0.9							1.0	
nr. of identified fragments	108	119	100	112	100	160	100	35	100	102	106	103	106	43	101	107	66	14	101	103	100

## Discussion

### 1. Chemistry of burned incense remnants

**Figure 8:**
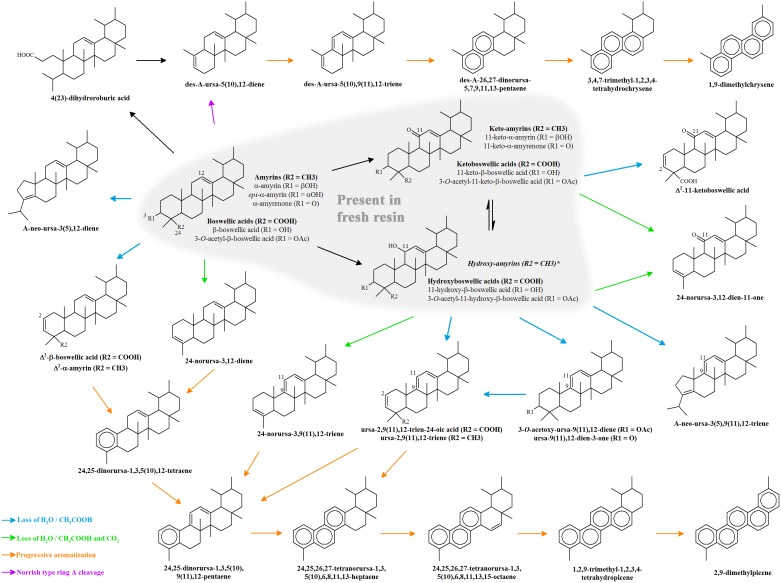
Summary of triterpenoid degradation reactions, demonstrated for ursane-type compounds. *Hydroxy-amyrins were not identified as such but are putative intermediates in the formation of dehydrated amyrins.

A prime objective was to identify the incense or incense mixture which has been burned in the late medieval funerary vessels from southern Belgium. Chemotaxonomic screening of the lipid extracts has provided unambiguous proof for frankincense, *viz.* the oleo-gum resin of *Boswellia* sp., but also revealed that the chemical signatures were greatly altered and differed almost completely from those of fresh frankincense (cf. Figure 2). An overview of all identified resin markers is given in Table 4. Original cembrane alcohols, tirucallic acids, boswellic and lupeolic acids were only recovered in trace amounts. Instead, 24-nortriterpenoids, amyrin derivatives and Δ^2^-triterpenoids were identified as major compounds. The overall good preservation state offers an excellent opportunity to investigate which chemical transformations have occurred during frankincense burning or during burial. A proposal for degradation pathways of ursane type compounds is presented in Figure 8. It should, however, be noted that the order of the separate degradation steps may not be fixed. Certain degradation products can still be linked to the original resin as will be seen below.

**Table 4:** Summary of major resin markers identified in samples R2, R9, Q10, Q11, Q12, L14 and L15. A full list of all individual compounds can be found in Table S1.

Resin	Resin markers	R2	R9	Q10	Q11	Q12	L14	L15
Frankincense	(keto)boswellic acids		tr		tr			
	24-nortriterpenoids^a^	+++	+++		+++	+++	+++	+++
	Δ^2^ boswellic acids^b^	++	++		++	++		
	4/5-ring PAHs	+	+		+	+	+	+
	tirucallic acids		tr		tr			
	cembranoids^c^				tr		tr	tr
Pinaceae tar	dehydroabietic acid, retene, etc.	tr	tr	+++			tr	tr
Cupressaceae/Podocarpaceae	ferruginol, totarol				+	+		

a 24-nortriterpenoids include 24-norursa-3,9-diene, 24-norursa-3,9(11),12-triene and corresponding oleananes.

b Δ^2^ boswellic acids include Δ^2,12^ dienes, Δ^2,9(11),12^ trienes and 11-keto-Δ^2,12^ dienes.

c Cembranoids include cembrene, serratol and incensol.

Amyrins, their acetates and oxidized forms are widespread phytochemicals and are produced by many higher plants [67], including *Boswellia* sp. [20], [26]. By contrast, 24-nortriterpenoids such as Δ^3,12^-ursadienes and Δ^3,9(11),12^-ursatrienes are much more specific. They were already identified in *Boswellia* resin pyrolysates and are produced from boswellic acids and their corresponding acetates (Figure 8). They are formed through a combined decarboxylation and dehydration (resp. deacetoxylation) in which the carboxylic acid at C-24 plays a crucial role as favored leaving group [22]. Apart from natural degradation, they may also be formed in the hot injector of the gas chromatograph [20], [26]. Analyses of modern frankincense, however, demonstrate that the formation of these analytical artifacts is rather limited (cf. peaks 9–10 in Figure 2a). Therefore, their high abundance in the archaeological residues (peaks 7–10 in Figure 2b, 2d) suggests that they represent markers for degraded frankincense.

Δ^2^-boswellic and Δ^2^-lupeolic acids constitute another group of diagnostic compounds, which are identified here for the first time. They were not detected when analyzing the modern reference resins and may be considered as first stage degradation products of boswellic and lupeolic acids, *i.e.* they are most likely formed by dehydration of the 3α alcohol functionality (Figure 8) as is the case for amyrins [54]. Fortunately, the diagnostic carboxylic acid group on C-24 is preserved which makes these compounds suitable as univocal biomarkers for degraded frankincense. Furthermore, the presence of ring A contracted neotriterpenoids and Δ^9(11),12^ triterpenoids (Figure 8) testifies that dehydration processes have indeed taken place. Neotriterpenoids are formed from amyrins through a Nametkin rearrangement [54] and the Δ^9(11),12^ double bond constitutes the dehydrated form of Δ^12^ triterpenoids with an alcohol functionality on C-11 such as 11-hydroxyboswellic acids which are naturally present in frankincense [55]. Dehydration reactions may have taken place during mild pyrolysis or during prolonged contact with desiccants such as charcoal.

Of particular interest are also the polyunsaturated hydrocarbons and PAHs which were present in minor amounts. To date, these compounds have only been identified in sedimentary rocks and lake or deep-sea sediments of geological age, *e.g.* [52], [53], [55], [56], [68]. Tetracyclic hydrocarbons such as des-A-ursa-5(10),12-diene are formed by a Norrish type cleavage of the A ring [69] and, like pentacyclic oleanane and ursane compounds, may undergo a series of dehydrogenation, demethylation and progressive aromatization reactions to form PAHs such as dimethylchrysene and dimethylpicene (Figure 8) [53]. These types of reactions also act upon abietane and pimarane diterpenoids during pine tar or pitch production (cf. diterpenoid profile of sample Q10; [58], [70]) and during pine wood combustion [71]. At mild pyrolytic conditions, *viz.* temperatures between 100–200°C, abietic acid dehydrogenates to its more stable and monoaromatic derivative dehydroabietic acid [58]. Further thermal treatment of the tars at temperatures above 300°C initiates decarboxylation, dealkylation and aromatization reactions, which generate partially and fully aromatic hydrocarbons such as retene and pimanthrene [71]–[73]. Radical pathways leading to the formation of more toxic and higher molecular weight PAHs, e.g. benzo[a]pyrene and benzofluoroanthenes, only proceed at temperatures in excess of 400°C [72]. Although frankincense triterpenoids may not necessarily behave in the same way as pine wood diterpenoids, the low abundance of the polyunsaturated hydrocarbons and PAHs and the absence of higher molecular weight PAHs seems to suggest that these funerary pots have undergone only mild pyrolytic conditions.

Despite the trace amounts of original frankincense constituents, further identification to species-level is not impossible. Inter-species variation in chemical composition was verified by analyzing commercial resins of *B. carterii*, *B. papyrifera*, *B. sacra* and *B. serrata*. Our results are summarized in Table 2 and correspond well to published data [20], [26], [46]. However, many identification criteria that apply for fresh frankincense, such as the ratio of 3-*O*-acetyl-11-keto-β-boswellic acid to 11-keto-β-boswellic acid and the percentages of lupane or tirucallane compounds [45], [46], cannot be used for the ancient residues. For instance, relative concentrations of amyrins, boswellic acids, lupeolic acids and tirucallic acids may change during pyrolysis or burial because of different degradation kinetics. Nevertheless, the species *B. frereana*, *B. neglecta* and *B. rivae* can be excluded because these resins do not contain boswellic acids in significant amounts [26], [74]. Furthermore, diterpenoids have been found to be resistant to chemical changes in pyrolysis experiments [26] and were recovered in traces amounts in samples Q11, L14 and L15. According to the deconvoluted diterpenoid profiles in the commercial resins, the dominance of serratol in the archaeological samples corresponds to *B. sacra* or *B. serrata* and thus excludes *B. papyrifera* and *B. carterii*. However, these identifications are very preliminary as only one sample from each species was analysed. Therefore, we advocate further research on these diterpenoid constituents to assess inter- and intra-species variability (*e.g.* differences in age, soil type, season and microclimate among all relevant *Boswellia* species).

Among the residues that displayed a major frankincense signature, traces of other potential incense ingredients were also retrieved. For instance, samples R2, R9, Q10, L14 and L15 displayed traces of pine tar. These signatures could, however, also relate to an earlier vessel use (see below). More interesting are the biomarkers ferruginol, totarol and corresponding ketones which were identified in samples Q11 and Q12. They could derive from the Cupressaceae (*e.g. Tetraclinis*, *Juniperus*, *Cupressus*) and Podocarpaceae (*e.g. Podocarpus*) family as well as from *Cedrus atlantica* [60]. Podocarpaceae and *Cedrus Atlantica* are unlikely sources as these conifers are native to the southern hemisphere and the Atlas mountains in Morocco and Algeria, respectively. Sandarac resin, derived from *Tetraclinis*, can also be excluded as this resin should also contain free diterpenoids such as sandaracopimaric acid and acetoxy agatholic acid, even after aging or pyrolysis [75]. From the Cupressaceae family, juniper (*Juniperus communis* L.) is the only species that occurred in Belgium during the middle ages although it was probably rare in the study area as it prefers poor, sandy soils like the coastal dunes and the Campine region. Moreover, juniper berries have been commonly used in medieval Europe as a fragrant material [76] and contain totarol as the major diterpenoid constituent [77].

### 2. Function of funerary vessels

Funerary ceramics were common in Roman tombs and were used as a container of cremated remains or food offerings. With the Christianization of Europe, the tradition of grave gifts, particularly in its northwestern regions, was gradually abandoned with a total disappearance by the 8^th^ century. In the 12^th^ century, grave gifts reappeared, however with a different functionality. Medieval funerary ceramics in northwestern Europe are generally interpreted as incense burners, and in some rare cases as containers for holy water, based on their appearance in historical manuscripts, *e.g.* Durandus’ *Rationale Divinorum Officiorum*, and iconographic sources, *e.g.* miniatures from the Book of Hours (Figure S2) [30], [32]. In the Belgian contexts, however, holy water was not kept in ovoid pots or pitchers but rather in recipients which have a more open form and are made of stoneware, glass or tin [31], [78]. Our data on charcoal and organic residues present, for the first time, clear material proof for the interpretation that funerary vessels in northwestern Europe have been primarily used as censers. Additionally, a single vessel from a domestic context (sample N13) seems to have been used for food-related purposes. These assertions will be clarified below.

The organic remains from the studied pots from Rebecq, Quaregnon and Liège show not only univocal evidence for burned frankincense (except for sample Q10); some of the vessels also contain potential evidences that frankincense was not used in a pure grade but has been admixed with other scents such as juniper and possibly pine tar. The fact that the juniper biomarkers only occurred in trace mounts indicates that frankincense must have been the dominant resin compound. Despite a general emphasis on both spiritual and material purity in this period, frankincense was expensive and cutting with more commonly available scents such as flowers, herbs and fragrant woods would have lowered the price. The scant literary evidences of incense mixtures date from later periods but let believe that diluting frankincense has indeed been common practice for some time, whether it was officially allowed or not. In 1571, Pope Pius V allowed that Peru balsam (*Myroxylon pereirae*) could be used for incense and later in 1606, the *Caeremoniale Episcoporum* decreed that frankincense should be used in pure grade or, when admixed with other materials, should be the major incense constituent [15]. Today, incense in the catholic church is composed of various ingredients including frankincense, myrrh, styrax, storax, sandalwood, cloves and lavender (cf.www.threekingsproducts.com) [2]. How exactly frankincense was brought to Belgium cannot be stated with great certainty. A possibility is that the resin was imported by crusaders who used to bring many luxury goods from the Holy Land to Europe.

Charcoals from the pots from Liège and Huy and from two pots from Rebecq, show a rather high number of taxa, probably reflecting the collection of charcoal from domestic fireplaces. This interpretation is supported by the find in sample R1 of a chunk of clay fused with the ashes of a fire into a glassy fuel ash slag, typical for high temperature processes and furnaces used in artisanal activities. The recovery of charcoal from domestic contexts is further supported by the presence of charred seeds and bones in perforated pots (L14, L17) from the cathedral of Liège. These charred remains include seeds or fragments of seeds from *Pisum sativum*, unidentified Fabaceae and cereals as well as bone fragments from unidentified mammalian bones and herring vertebrae (Q. Goffette and S. Preiss, personal communication). In contrast, in the 7 pots recovered from a single pit in the central nave of the parish church of Saint-Géry (Rebecq), only charcoal from beech has been found. This might be an indication for intentionally produced charcoal as this is likely to result in a monospecific assemblage [79]–[81].

Almost all perforated pots in this study were recovered from burials within ecclesiastical buildings (Table 1) and thus belonged most likely to clergy members or nobleman [82]. Nevertheless, perforated pots can also be found in outdoor burials, sometimes in graves without a coffin, suggesting that the pots were not only reserved for the elite. These hypotheses remain unverified, however, and pose appealing questions for future research such as the comparison of the incense composition of funerary pots from indoor and outdoor burials. The perforated pot from Namur (sample N13) stands out as the only one recovered from a domestic context. The presence of heated ruminant fats in this sample is clearly not consistent with an incense-related function but rather suggests that the vessel has been used for preparing foods (see below).

The funerary pots relate to a cultic use with a profound spiritual meaning as incense and its use in funerary rites are deeply intertwined in Christian worship, dating back even to the earliest periods of Christianity. Yet, its symbolic meaning has changed through history. Early Christian sources such as Tertullian, whilst repudiating the use of incense in worship, affirm that it was used as embalmment *ad solatia sepulturae*, *i.e.* to reduce the odours of decomposing bodies [15], [83]. Later on, it became associated with the hope of resurrection and was applied in triumphal funeral processions like that of Peter of Alexandria. While embalming and censing at funerals persisted in the Middle Ages, the sweet scents of frankincense were also conceived, together with holy water, as a repellent against the foul stench of evil spirits [29], [83]. Furthermore, late medieval manuscripts by Beleth, Sicard and Durandus state that frankincense embodies the good deeds of the departed as well as the prayers owed to them and that the charcoal marks the sacred character of the earth in which they are buried [29], [33].

### 3. Vessel recycling

The fact that most perforated vessels are ordinary domestic ceramics and have been pierced after firing suggests that the vessels had an earlier use-phase prior to their use as censers. We attempted to find chemical evidence for this by investigating not only surface residues but also absorbed organic residues inside the ceramic fabric whenever possible. The latter are known to be depleted in intrusive soil lipids [50], [84], [85], thus avoiding uncertainty in the interpretation of potentially food-derived lipids that were detected in some of the residues (*viz.* traces of fatty acids, alkanols and alkanes).

The ovoid pot from Namur (N13) represents the clearest evidence for a domestic provenance. Not only was the pot found in a cesspit from an urban domestic site, it also contained clear markers of heated ruminant fats suggesting its use as a cooking vessel. Durand [35] has already postulated, based on visible traces of use and iconographic sources, that perforated ceramics had been used for food preparation prior to their application in funerary rites. The vessel from Namur (N13), however, lacks clear evidence for incense burning, which possibly indicates that the vessel was pierced to adapt it for use in funerary rituals but has never been actually used for that purpose. Another hypothesis is that the vessel has been recycled as a lamp and that an animal fat such as ruminant tallow has been used as fuel. It is well established that animal fats as well as various plant oils have been widely employed as illuminants in Roman and medieval periods [86]–[90].

Furthermore, the pine tar signature of vessel Q10 constitutes another possible indication for vessel recycling. Pine tar and pitch have been widely used to coat ceramics to make them impermeable for liquids such as wine, oils or garum [91], with the earliest evidence dating back to the 7^th^ century BC [70]. While pitch coatings were very common in classical antiquity [92], [93], it is not clear if this technique was still used in late medieval ceramics due to a lack of chemical research. Nevertheless, pine pitch continued to be used as a waterproofing material which is evidenced by analyses of contemporary naval timbers like that of the Mary Rose, the flagship of king Henry VIII [70], [94], and the Bremer Kogge [95]. Moreover, there are evidences of late medieval pitch production sites like that of Ruppersdorf in Germany [96]. Possibly, pine tar has also been used as coating for this funerary vessel. In that case, however, it must refer to an earlier use-phase involving liquid handling or storage since the perforations suggest that it was used as a censer. The absence of frankincense markers might be due to the fact the sample has been extensively washed following excavation.

Absorbed residues from vessels R2 and R9 did not contain lipids which could be related to a vessel use other than incense burning, although markers for pine tar in the corresponding surface residues might relate to an earlier use associated with liquid handling or storage. Alternatively, pine resin or rosin could also have been used as a minor incense ingredient (see above). Minor traces of pine tar were also found in samples Q11, L14 and L15.

The act of vessel recycling and the use of pots of inferior quality in particular, are in stark contrast with the precious nature of the frankincense. This apparent discrepancy remains enigmatic, however, and one can but adhere to a few certainties, namely (i) that the vessels are indeed of inferior value as more elaborate wares of higher quality (other techniques, typology, etc.) have been made in this period, (ii) that they were not only reserved for the elite, for the clergy nor for any other social class (*cf. supra*) and (iii) that the pots were used as soon as the corpse is placed on the bier as evidenced by iconographic sources (Figure S2) [31]. Questions remain as to whether the pots and the frankincense might have been provided by separate entities, *viz.* the family and church officials, respectively? Or could the use of inferior pots perhaps be explained by a dichotomy between the ban on grave gifts imposed by the catholic church and a general desire to provide the departed with religious symbols such as frankincense? In any event, the appearance of funerary incense pots from the 11–12^th^ century onwards indicates a growing concern about a person’s fate, a life after death, and how the latter may be improved. The fear of an all-destroying Death and purgatory further proliferated in the 14^th^ century when the Black Death traumatized the European population.

## Conclusion

Incense burning in religious and domestic contexts has existed since the beginnings of our civilization. Yet, few chemical analyses have been performed on remnants of this widespread ritual, which in part may be due to the misconception that incense is a fairly volatile substance leaving no traces after burning. Nevertheless, certain resins such as frankincense contain a substantial amount of non-volatile components which are well preserved in the archaeological record. Heating in contact with ceramics or charcoal induces myriad chemical transformations but, fortunately, not necessarily to such an extent that the original incense material cannot be recognized anymore. Our data from late medieval funerary censers from southern Belgium allowed to study these chemical changes. 24-nortriterpenoids were abundant compounds in most samples and are formed from boswellic acids during pyrolysis. Novel compounds, namely Δ^2^-boswellic acids, were identified based on mass spectral data and retention characteristics. These compounds represent dehydration products which may have formed during mild pyrolysis or during prolonged contact with desiccants such as charcoal. Furthermore, small amounts of polyunsaturated and fully aromatic hydrocarbons and the absence of toxic PAHs such as benzo[a]pyrene are indicative for mild pyrolysis conditions. Unaltered frankincense compounds such as boswellic acids, tirucallic acids and cembrane type alcohols were only present in trace amounts. Particularly the latter were nevertheless instructive to indicate *Boswellia sacra* and *Boswellia serrata* as possible botanical sources. Still, further research is needed to resolve these diterpenoid alcohols and to verify the proposed species-specific identification criteria following a thorough assessment of inter- and intraspecies variability.

The perforated pots from southern Belgium, apart from providing an occasion to study well preserved burned frankincense remains, also constitute an intriguing find category which, despite its widespread occurrence in western Europe, has not been investigated in depth by advanced scientific techniques. We report herein the first material proof of their function as censers and, in one case, for food preparation. Chemical analyses evidenced that frankincense was used as the major incense ingredient and that small amounts of juniper and possibly pine resin have been added, most likely to reduce the cost. Anthracological analysis revealed that charcoal was most likely recycled from domestic fires in two thirds of the cases, whereas the remaining vessels might contain intentionally produced charcoal. Except for the vessels that were perforated before firing, the vessels themselves were probably not primarily made for censing as we found indications that some of them have been used for alimentary purposes. Our results have demonstrated the high potential of chemical analyses to identify incense mixtures in perforated funerary pots but further research is needed to assess variability on a larger interregional scale.

## Supporting Information

Figure S1 **Structures of major diterpenoids and triterpenoids cited in the text.**
(TIF)

Figure S2 **Miniature from “Petites Heures” of Jean de Berry displaying the use of pots during a funeral service (National library of France, Paris, Latin 18014, fol. 134v, c. 1385–1390).**
(JPG)

Table S1 **List of all identified molecules with mass spectral information and retention characteristics.**
(XLSX)

File S1 **Identification of Δ^2^-derivatives of boswellic acids based on mass spectral data and retention characteristics.**
(DOCX)
