# Supplementary figures and images for: Holy Smoke in Medieval Funerary Rites: Chemical Fingerprints of Frankincense in Southern Belgian Incense Burners
Source: PLoS One. 2014 Nov 12;9(11):e113142. doi: 10.1371/journal.pone.0113142 (PMC4229304; doi:10.1371/journal.pone.0113142)

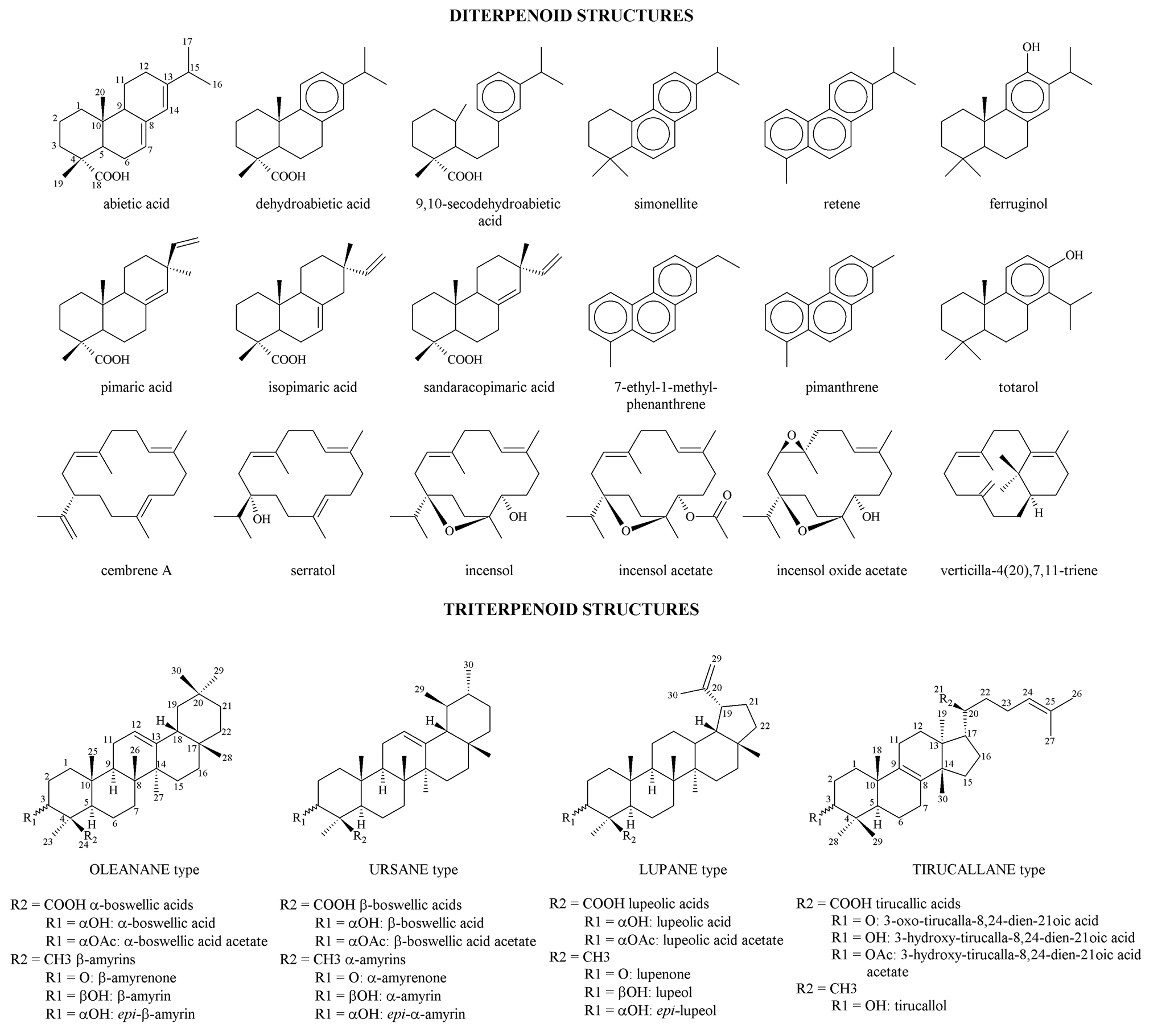

Supplement: Figure S1 — Structures of major diterpenoids and triterpenoids cited in the text. (TIF) [file pone.0113142.s001.tif]

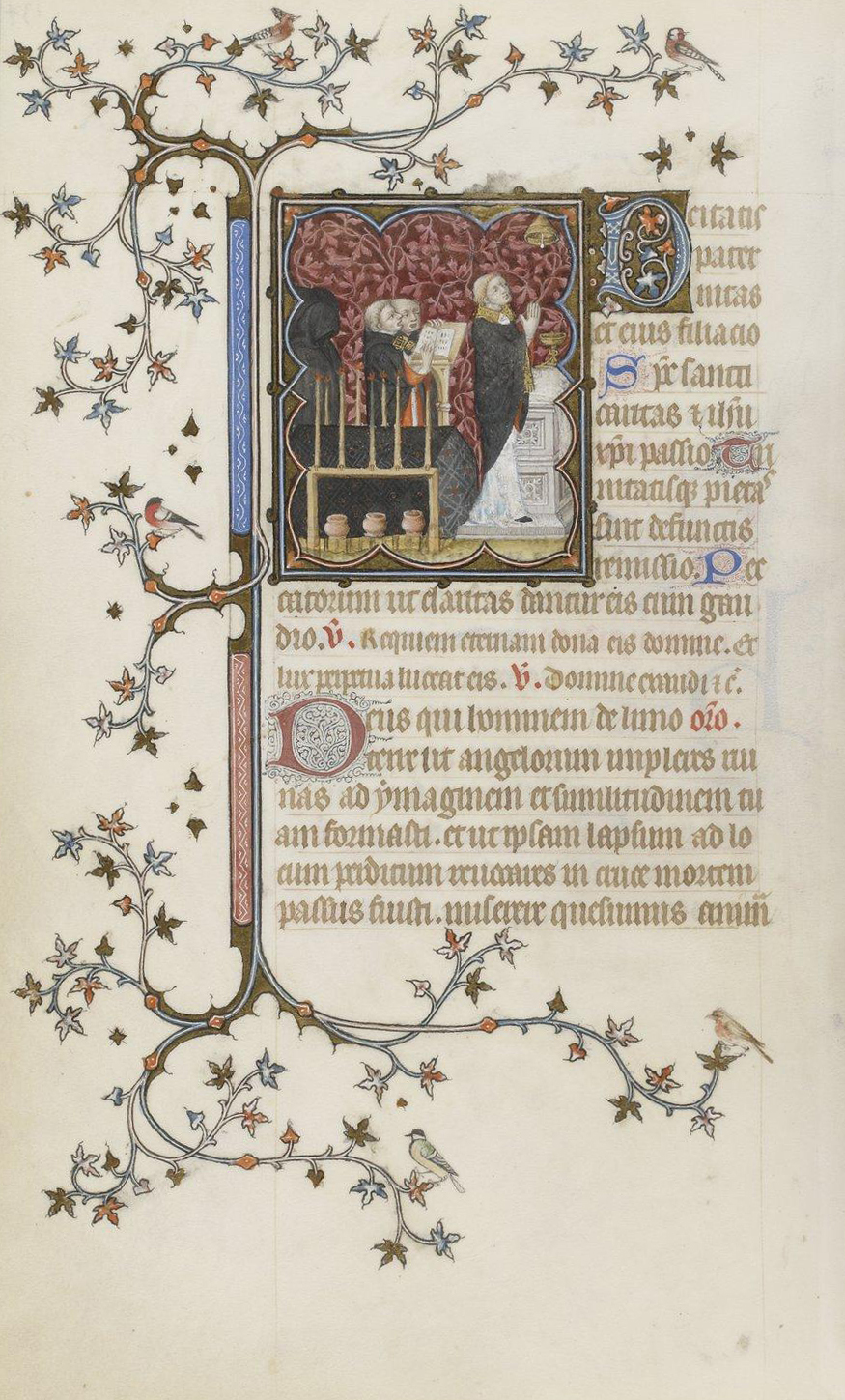

Supplement: Figure S2 — Miniature from “Petites Heures” of Jean de Berry displaying the use of pots during a funeral service (National library of France, Paris, Latin 18014, fol. 134v, c. 1385–1390). (JPG) [file pone.0113142.s002.jpg]
