# Supplementary material for: Holy Smoke in Medieval Funerary Rites: Chemical Fingerprints of Frankincense in Southern Belgian Incense Burners
Source: PLoS One. 2014 Nov 12;9(11):e113142. doi: 10.1371/journal.pone.0113142 (PMC4229304; doi:10.1371/journal.pone.0113142)
Supplement: File S1 — Identification of Δ2-derivatives of boswellic acids based on mass spectral data and retention characteristics. (DOCX) [file pone.0113142.s004.docx]

**Identification of Δ^2^-derivatives of boswellic acids based on mass spectral data and retention characteristics**

The mass spectral fragmentation pattern of oleanane and ursane triterpenoids, and boswellic acids in particular, is well attested [1-4] and enabled to identify a number of novel Δ^2^ derivatives of boswellic acids. The mass spectra (figure 4a–4d) and retention characteristics of these compounds (supplementary table S2) are here explained in more detail. For clarity, the compounds that are associated with the mass spectra of figure 4 are abbreviated here by Roman numerals.

1. *Oleana-2,12-dien-24-oic acid (compound* ***I****, figure 4a) and ursa-2,12-dien-24-oic acid (compound* ***II****, figure 4b).*

The mass spectra of both compounds show clear features of a Δ^12^-unsaturated oleanene or ursene as revealed by the base peak of *m*/*z* 218. This fragrment is due to a retro-Diels-Alder (rDA) fragmentation in which ring D and E are retained [2]. Further loss of a methyl group leads to the formation of the *m*/*z* 203 ion. The intensity of this ion is higher for Δ^12^-oleanenes [3] because of the higher stability of a tertiary carbenium ion which may be formed by loss of the C-28, C-29 or C-30 methyl group. In Δ^12^-ursenes, loss of the C-28 methyl group is the only fragmentation that leads to a tertiary carbenium ion while loss of the C-29 or C-30 methyl would lead to a less stable secondary carbenium ion. Therefore, compound **I** and compound **II** correspond to a Δ^12^-oleanene and Δ^12^-ursene structure, respectively.

A medium intensity peak at *m*/*z* 292 indicates that the structures of compound **I** and **II** contain a carboxylic acid at C-24 and thus must be a derivative of boswellic acid. The *m*/*z* 292 ion is formed by rDA fragmentation with retention of ring A and B. It is also present in the mass spectra of boswellic acid or boswellic acid acetate, where its formation involves an additional loss of a TMSOH or AcOH group, respectively. The *m*/*z* 292 fragment can further loose the carboxylic acid group to form an *m*/*z* 175 ion. An intense peak at *m*/*z* 73 further indicates the presence of a TMS group. The molecular weight, which can be deduced from the [M–15]^+^ and [M]^+•^ fragments (*m*/*z* 495 and 510, respectively), indicates a loss of 90 mass units when compared to boswellic acid (C_36_H_64_O_3_Si_2_, MW 600). This corresponds to the loss of a TMSOH group, *in casu* the derivatised alcohol group on C-3. Therefore, compounds **I** and **II** most likely correspond to oleana-2,12-dien-24-oic acid and ursa-2,12-dien-24-oic acid, respectively.

This identification is further sustained by the retention characteristics of these compounds. It is well attested that oleanane type compounds elute before ursane type equivalents on apolar GC columns such as HP-5MS [3-5]. This is also the case for compounds **I** and **II** as can be observed in figure 2b and 2c. Furthermore, compounds **I** and **II** exhibit slightly earlier retention times when compared to α- and β-boswellic acid, respectively. The difference in retention time between Δ^12^-oleanenes (compound **I** and α-boswellic acid) and Δ^12^-ursenes (compound **II** and β-boswellic acid) amounts to ~1 min (constant heating rate of 4 °C min^-1^).

1. *Ursa-2,9(11),12-trien-24-oic acid (compound* ***III****, figure 4c)*

The mass spectrum of compound **III** shows similarities with mass spectra of Δ^9(11),12^-unsaturated oleanene and ursene structures such as 24-norursa-3,9(11),12-triene (C_29_H_44_, MW 392). These compounds lack any rDA fragments due to the conjugated double bond system. Instead, they are characterized by a very intense [M–15]^+^ and [M]^+•^ peak and medium intensity peak at *m*/*z* 255, which is due to a cleavage across the B ring with retention of ring C, D and E [4]. A similar fragmentation can occur due to cleavage across the D ring yielding a fragment containing the A, B and C ring, *e.g.* ion *m*/*z* 239 in 24-norursa-3,9(11),12-triene. The mass spectrum of compound **III** also shows a peak at *m*/*z* 73 indicating the presence of a TMS group. Although both alcohol (OTMS) and carboxylic acid (COOTMS) groups would yield such an ion, a carboxylic acid group is the more likely candidate in view of the high molecular weight. In fact, the molecular ion [M]^+•^ at *m*/*z* 508 is just two mass units lower than that of Δ^2^-boswellic acid (see above, figure 4a, 4b). Therefore, the compound **III** may correspond to a Δ^2,9(11),12^-boswellic acid. Cleavage across the D ring would thus yield an *m*/*z* 355 ion, which is indeed observed in the mass spectrum. Furthermore, the *m*/*z* 375 ion may be explained by the loss of the carboxylic acid group and the C-25 methyl group yielding a resonance stabilized fragment containing four conjugated double bonds. The *m*/*z* 269 ion may represent a fragment that results from an alternative ring B cleavage as depicted in figure 4c.

Further proof is given by the fact that compound **III** elutes just before Δ^2^-boswellic acids. Similar patterns are observed for Δ^3,9(11),12^ and Δ^3,12^ unsaturated 24-nortriterpenoids, *i.e.* the Δ^3,9(11),12^ trienes elute just before the Δ^3,12^ dienes (see supplementary figure S4). As oleanane type compounds elute before their ursane equivalents (see above), compound **III** (peak 6 in figure S4) corresponds to ursa-2,9(11),12-trien-24-oic acid while peak 5 corresponds to oleana-2,9(11),12-trien-24-oic acid.


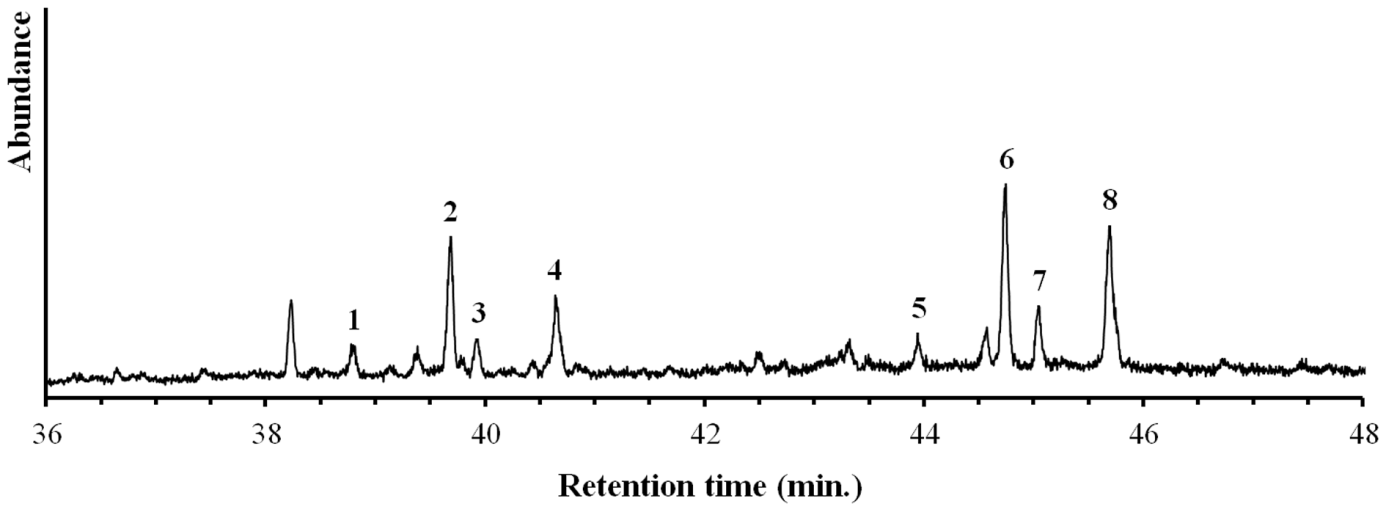


**Figure S4.** Summated extracted ion chromatograms (*m*/*z* 292, 378, 380 and 508) of sample Q11, showing the presence of 24-noroleana-3,9(11),12-triene (**1**), 24-norursa-3,9(11),12-triene (**2**), 24-noroleana-3,12-diene (**3**), 24-norursa-3,12-diene (**4**), oleana-2,9(11),12-trien-24-oic acid (**5**), ursa-2,9(11),12-trien-24-oic acid (**6**), oleana-2,12-dien-24-oic acid (**7**) and ursa-2,12-dien-24-oic acid (**8**). Diagnostic ion fragments were chosen as follows: [M-14]^+^ = *m*/*z* 378 (**1**,**2**), [M-14]^+^ = *m*/*z* 380 (**3**,**4**), [M]^+•^ = *m*/*z* 508 (**5**,**6**) and rDA fragment *m*/*z* 292 (**7**,**8**).

1. *11-keto-ursa-2,12-dien-24-oic acid (compound* ***IV****, figure 4d)*

The mass spectrum of compound **IV** shows features which are also observed for 11-keto-boswellic acids [1]. These include a rDA fragment with *m*/*z* 232 and an *m*/*z* 273 ion which is due to a McLafferty rearrangement involving the migration of the C-1 hydrogen atom to the C-11 oxygen atom followed by the homolytic cleavage of the A+B ring. Further rearrangement of the *m*/*z* 273 ion leads to the formation of an *m*/*z* 135 ion [1]. The molecular weight, which can be deduced from the [M–15]^+^ and [M]^+•^ fragments (*m*/*z* 509 and 524, respectively), indicates a loss of 90 mass units when compared to 11-keto-boswellic acid (C_36_H_62_O_4_Si_2_, MW 614). This corresponds to the loss of a TMSOH group, *in casu* the alcohol group at C-3. Furthermore, the *m*/*z* 407 and *m*/*z* 480 ions may be explained by a homolytic cleavage of the carboxylic acid group and by the cleavage of a neutral CO_2_ molecule, respectively. Therefore, compound **IV** most likely corresponds to 11-keto-ursa-2,12-dien-24-oic acid.

This is further sustained by comparing the retention time of compound **IV** and 11-keto-β-boswellic acid. The difference in retention time amounts to ~1 min (constant heating rate at 4 °C min^-1^), which is similar to the difference in retention time between Δ^2^-boswellic acids and their corresponding boswellic acids (see above).

**References**

[1] Basar, S. 2005. Phytochemical Investigations on Boswellia Species. PhD thesis, University of Hamburg.

[2] Budzikiewicz, H., Wilson, J.M., Djerassi, C. 1963. Mass Spectrometry in Structural and Stereochemical Problems. XXXII. Pentacyclic Triterpenes. Journal of the American Chemical Society 85, 3688–3699.

[3] Mathe, C., Culioli, G., Archier, P., Vieillescazes, C. 2004. Characterization of archaeological frankincense by gas chromatography-mass spectrometry. Journal of Chromatography A 1023, 277–285.

[4] Ten Haven, H.L., Peakman, T.M., Rullköter, J. 1992a. Early diagenetic transformation of higher-plant triterpenoids in deep-sea sediments from Baffin Bay. Geochimica et Cosmochimica Acta 56, 2001–2024.

[5] Regert, M., Devièse, T., Le Hô, A.S., Rougeulle, A. 2008. Reconstructing ancient Yemeni commercial routes during the Middle Ages using structural characterization of terpenoid resins. Archaeometry 50, 668–695.
